# Which infancy growth parameters are associated with later adiposity? The Cambridge Baby Growth Study

**DOI:** 10.1080/03014460.2020.1745887

**Published:** 2020-05-20

**Authors:** Ken K. Ong, Tuck Seng Cheng, L. Olga, P. M. Prentice, C. J. Petry, I. A. Hughes, D. B. Dunger

**Affiliations:** a MRC Epidemiology Unit, University of Cambridge, Cambridge, UK;; b Institute of Metabolic Science, University of Cambridge, Cambridge, UK;; c Department of Paediatrics, University of Cambridge, Cambridge, UK;; d North Middlesex University Hospital NHS Trust, London, UK

**Keywords:** Infancy, weight gain, growth, adiposity, obesity, prediction

## Abstract

**Background:** Highly consistent positive associations are reported between infancy growth and later obesity risk. However, it is unclear whether infancy growth parameters beyond body weight add to the prediction of later obesity risk.

**Aim:** To assess whether infancy length and skinfold thicknesses add to infancy weight in the prediction of childhood adiposity.

**Subjects and methods:** This analysis included 254 children with available data on infant growth from birth to 24 months and childhood adiposity at age 6–11 years measured by DXA. Multilevel linear regression was used to examine the predictors of childhood percent body fat (%BF), with adjustment for sex and age at follow-up visit.

**Results:** Birth weight and weight gain (modelled as changes in z-score) between 0–3 months and 3–24 months showed independent positive relationships with childhood %BF. The addition of gains in infant length and skinfolds between 0–3 months, but not 3–24 months, improved overall model prediction, from 18.7% to 20.7% of the variance in childhood %BF (likelihood ratio test, *p* < 0.0001), although their independent effect estimates were small (infant length gain: negative trend, partial R-square 0.6%, *p* = 0.2; skinfolds: positive trend, 1.3%, *p* = 0.09).

**Conclusion:** Infancy length and skinfolds contribute significantly, but only modestly, to the prediction of childhood adiposity.

## Introduction

Highly consistent findings have been reported from longitudinal birth cohort studies on the relevance of rapid infancy growth to later obesity risk. Our original studies in the ALSPAC cohort showed that infants with faster weight gain went on to have larger weight, BMI, WC and fat mass at age 5 years (Ong et al. 2000). While such positive association “might be expected”, it debunked the notion held by many, including health professions and families, that rapid weight gain in infancy is simply “puppy fat” and without lasting consequences. Our follow-up of the ALSPAC cohort showed that rapid infancy weight gain also predicts higher adiposity and insulin resistance at age 8 years (Ong et al. 2004), and in due course earlier puberty timing (Ong et al. 2009), which in turn is a risk marker for many later life diseases and earlier mortality (Charalampopoulos et al. 2014; Day et al. 2015). Since that study, similar findings have been replicated in many other settings, and have been shown to apply to infants of low as well as normal and high birth weight (Druet et al. 2012; Ong and Loos 2006). In a recent systematic review, Woo Baidal et al. found that 41 out of 42 studies reported a significant positive association between infant weight gain, or infant body weight, and risk of childhood overweight or obesity (Woo Baidal et al. 2016).

Our original ALSPAC report did not explore the shape of the association between infancy weight gain and later obesity (Ong et al. 2000). Alongside our primary analyses of infancy weight gain as a continuous variable, we reported the risks relating to infants who gained ≥ +0.67 in their weight z-score, as such infants are easily clinically recognised by upwards weight centile crossing on standard growth charts. Subsequently, we did examine the shape of this association using pooled individual-level data from 10 cohort studies, including ALSPAC (Druet et al. 2012). We found that the risk of childhood overweight or obesity increased linearly across most of the observed range of infant weight gain, but increased more steeply with rates of weight gain > + 1.33 z-scores, a threshold that identifies a minority of infants (∼10%) who cross upwards through >2 weight centile bands. This “inverted-J” shape of association is similar to that reported for many other epidemiological associations, such as between adult BMI-to-mortality, blood glucose-to-diabetic retinopathy, or blood pressure-to-stroke, and underlies Geoffrey Rose’s principles of preventive medicine that advocate for the benefits of population-level reductions in risk factor levels, alongside individual-level approaches that define, identify and “treat” high-risk individuals (Rose 1993). Both population-level and individual-level approaches are of current interest in efforts to prevent childhood obesity (Blake-Lamb et al. 2016). Recent reports that UK infants are on average overnourished and overweight highlight the need to monitor and identify population-level determinants of excessive infant weight gain (Scientific Advisory Committee on Nutrition (SACN) 2018). To inform the latter individual-level approach, we showed that the addition of data on infant weight gain to data on maternal BMI and birthweight substantially improved the prediction of childhood overweight or obesity to levels with potential clinical utility (Druet et al. 2012), and this is a topic of ongoing interest (Daniels et al. 2015; Redsell et al. 2017; Lakshman et al. 2018; Paul et al. 2018).

With that in mind, there remain important questions regarding the association between infancy growth and later obesity risk. Foremost, and the main topic of this paper, is that almost all of the existing evidence relates solely to infant body weight as the exposure (Woo Baidal et al. 2016). It is important to ascertain whether other infancy growth parameters, beyond body weight, add to the prediction of later obesity risk. In particular, it seems intuitive to think that, alongside infant weight gain, faster gains in infant length would be protective for later obesity risk and, conversely, faster gains in measures of infant adiposity might exacerbate the risk of later obesity (Ong 2017). Such features likely impact on how parents and health professionals perceive the risks of overweight and obesity for individual infants, however, there is as yet (to our knowledge) no evidence published on this topic.

One major caveat to such childhood prediction studies needs to be considered – the choice of which outcome measure of childhood obesity might have a major influence on the findings. Definitions of childhood overweight and obesity that are based on body mass index (BMI) may be ‘biased’ by the positive association between childhood BMI and childhood height, and it is possible that their associations with infancy weight may therefore largely reflect tracking of lean body mass. Childhood triponderal mass index (TMI) has been proposed as an alternative measure, as this index has a stronger numerical correction for height (TMI = weight/height^3, compared to BMI = weight/height^2) and has indeed been shown to be better than childhood BMI as a marker of adiposity (Peterson et al. 2017). However, paradoxically childhood TMI performs less well than childhood BMI when it comes to prediction of subsequent measures of cardio-metabolic disease, including: Type-2 diabetes, hypertension and LDL cholesterol levels, and carotid intimal thickness (Wu et al. 2018). This is likely because BMI additionally captures an element of childhood height that is related to a rapid tempo of growth and puberty timing, which is a recognised trajectory to later disease (Elks et al. 2012; Day et al. 2015). Ideally, the choice of the optimal measure and threshold of childhood adiposity used to define childhood overweight and obesity would include a strong consideration of the strength and shape of their relationships to related disease outcomes, including not only later life cardio-metabolic diseases but also the many childhood co-morbidities of obesity that encompass metabolic, respiratory, musculo-skeletal, neurological and psychological domains (Lakshman et al. 2012). In the current absence of such data on disease relationships, in this study, we have chosen percent body fat as the primary outcome because (i) it allows consideration of the balance between fat and fat-free mass; (ii) it does not conceptually assume that all of the influence of childhood height needs to be removed; (iii) it is commonly used and its values are easily understood. We included continuous variation in childhood BMI as a secondary outcome, to allow comparison with previous studies. However, we deliberately refrained from defining any high threshold of adiposity as the outcome, because such definitions (e.g. above 95^th^ percentile) solely consider obesity as a statistically “abnormal” condition compared to growth references, and that approach ignores whether such thresholds are meaningful with regard to disease comorbidities (Scientific Advisory Committee on Nutrition (SACN) 2012).

## Subjects and methods

### Study population and design

The Cambridge Baby Growth Study (CBGS) recruited expectant mothers at the Rosie Maternity Hospital, Cambridge, UK between 2001–2009 for the study of pregnancy and postnatal determinants of early infancy growth and metabolism (Prentice et al. 2016). Inclusion criteria were mothers attending a single antenatal centre in Cambridge, UK. Exclusion criteria were mothers aged <16 years, or unable to give informed consent. During infancy, offspring weight, length, and skinfold thicknesses were measured at 0, 3, 12, 18 and 24 months by research nurses. A childhood follow-up visit was performed at age 5–11 years old.

### Birth measurements and infancy anthropometry

Infants’ birth weights were obtained from hospital records. Newborn (within first 8 days) length and skinfold thicknesses, and subsequent measurements at 3, 12, and 24 months of age were performed by three trained paediatric research nurses, using identical protocols for all cohorts. Weight was measured to the nearest 1 g using a SECA 757 electronic baby scale. Length was measured to the nearest 0.1 cm using an Infantometer (SECA 416). Skinfold thickness was measured in triplicate at four sites (triceps, subscapular, flank, quadriceps) on the left side of the body using a Holtain Tanner/Whitehouse Skinfold Calliper (Holtain Ltd). The inter-observer variability was assessed by repeated measurements in 8 infants; the absolute and relative technical errors of measurement were 0.7 cm and 0.9%, respectively, for length, and 1.4 mm and 12.6% for flank skinfolds.

### Childhood follow-up visit

At age 5–11 years old, children were invited to re-attend the research clinic. Between September 2013 and October 2018, we sent out 817 invitation letters to those who had previously attended the infancy phase of the study and met the following inclusion criteria: gestational age ≥34 weeks; attended the infancy study at ≥12 months old; current age 5–10 years; and exclusion criteria: previously withdrew from the study; genetic or congenital growth disorder; serious illness or death; use of high dose oral or inhaled glucocorticoid medication. These data were screened by accessing the National Health Service Information Centre for health and social care resource. A second letter was sent if no response was received after 1 month. Of the 817 eligible children and mothers, 365 responded and 285 consented and took part in the childhood follow-up (Supplementary Figure 1).

Children attended the hospital’s clinical research facility after an overnight fast. Height was measured using a wall mounted stadiometer; weight was measured using electronic scales to the nearest 0.1 kg in light clothing without shoes. A whole body dual energy x-ray absorptiometry (DXA) scan was performed to estimate total body fat mass; prior to 28 May 2015 we used the Lunar Prodigy machine (*n* = 180); subsequently by iDXA (*n* = 131), including 49 children who were scanned on both machines to provide comparative data.

Both the infancy [LREC Ref: 00/325] and childhood follow-up phases of the Cambridge Baby Growth Study [REC Ref: 08/H0302/47] received research ethics committee approvals. Written informed consent was provided by the child’s legal guardian at both infancy and childhood phases; written assent was also provided by the children at the childhood follow-up visit.

#### Statistical analyses

Age- and sex-appropriate z-scores were calculated for weight and length measurements according to current UK recommendations: measurements at birth were compared to the British 1990 growth reference (birth measurements were adjusted for gestational age at birth); infancy measurements were compared to the WHO 2006 growth standards (Scientific Advisory Committee on Nutrition (SACN) 2007). For each of the four skinfold thickness measurements, an internal z-score was calculated, using residuals from a linear regression model, adjusting for infancy age, and sex. Measurements at birth and 3 months were also adjusted for gestational age. Mean skinfold SDS was used in analyses.

Body composition data from the Lunar Prodigy and iDXA were harmonised using the following equation: [total fat mass from iDXA (kg) = total fat mass from Prodigy (kg) X 0.98–0.43 (kg)], as described by Watson based on overlapping measurements in 95 children aged 6-16 years, including 49 children from the current study (Watson 2018). The estimated total fat mass from iDXA was used to calculate body fat percentage (%BF) using the formula: total fat mass (kg)/body weight (kg) X 100.

Multilevel linear spline models (Howe et al. 2016) were used to derive individual-level infant growth velocities (change in z-score per month) at 0–3 months and 3–24 months in weight, length and skinfold thickness. A knot point at 3 months was chosen on visual inspection of mean weight z-scores in this cohort (Supplementary Figure 2). Rapid infancy weight gain was defined as ≥+0.67 change in weight z-score between birth to 24 months.

This analysis included 254 children with available data on infant weight and childhood DXA %BF (Supplementary Figure 1). To examine the predictors of childhood %BF, multilevel linear regression was used, with adjustment for sex and age at follow-up visit. The baseline model included birth weight z-score and changes in weight z-scores at 0–3 months and 3–24 months. Further models were computed by adding one potential predictor at a time in chronological order (i.e. gestational age, parity, length and skinfold thickness at birth, exclusive breastfeeding for 3 months, and changes in length and skinfold thickness at 0–3 and 3–24 months). Models with and without each predictor were compared using the likelihood ratio test. Model improvement was defined if both the likelihood ratio test *p* values <0.05 and a lower Akaike’s information Criterion (AIC) value were observed. Each predictor that led to a significant overall model improvement was retained in the next iteration of the model building process. Results are presented with effect estimates and partial R-squared values for those variables included in final models. Effect estimates for infant growth were scaled to a + 1 change in z-score during the displayed age period. The analyses were repeated with childhood BMI z-score as the outcome. Sensitivity analyses were performed (i) using rapid infancy weight gain in the baseline model, (ii) with further adjustment for pubertal status (yes/no), and (iii) with childhood lean mass index (total body lean mass/height squared, kg/m^2^) as the outcome. All analyses were conducted using Stata 15.1 (StataCorp. 2017. *Stata Statistical Software: Release 15*. College Station, TX: StataCorp LLC).

## Results

### Sample characteristics

The 254 included children had outcomes assessed at age 6–11 years (mean: 9.5 ± 1.0), of whom the majority were aged 8–11 years (*n* = 223, 87%). Compared to excluded children (*n* = 553), included children were more likely to be girls (45.8% vs. 55.5%, *p* = 0.012). No difference was observed in gestational age, parity, exclusive breastfeeding for 3 months, weight, length and skinfold thickness at birth, and changes in weight, length and skinfold thickness z scores at 0–3 and 3–24 months between excluded and included children (Table 1).

**Table 1.** Infancy characteristics of included children and excluded children.

	Included children (*n* = 254)	Excluded children (*n* = 553)	*p* Value
Sex (*n*, %)			0.012
Boys	113 (44.5%)	300 (54.3%)	
Girls	141 (55.5%)	253 (45.8%)	
Gestational age, weeks	40.0 ± 1.2	40.0 ± 1.3	0.492
Parity	1.8 ± 0.9	1.8 ± 0.8	0.384
Exclusive breastfed for 3 mo (*n*,%)			0.164
No	127 (51.4%)	305 (57.0%)	
Yes	120 (48.6%)	230 (43.0%)	
Birth weight, kg	3.54 ± 0.46	3.54 ± 0.50	0.893
Birth weight z-score	0.13 ± 0.95	0.11 ± 0.95	0.753
Change in weight z-score*			
0–3 mo	−0.04 ± 0.35	−0.04 ± 0.33	0.725
3–24 mo	0.03 ± 0.04	0.03 ± 0.05	0.614
Length at birth, cm	51.5 ± 2.50	51.4 ± 2.48	0.764
Length z-score at birth	0.42 ± 1.16	0.39 ± 1.18	0.695
Change in length z-score*			
0–3 mo	−0.09 ± 0.41	−0.11 ± 0.38	0.406
3–24 mo	0.01 ± 0.05	0.01 ± 0.05	0.235
Skinfold thickness z-score at birth	−0.06 ± 0.88	0.01 ± 0.87	0.267
Change in skinfold z-score*			
0–3 mo	0.02 ± 0.35	0.00 ± 0.34	0.416
3–24 mo	0.00 ± 0.05	0.00 ± 0.06	0.932

Values are mean ± SD unless indicated otherwise.

*Derived individual-level growth velocities (change in z-score per month).

At this follow-up visit, mean (±SD) weight was 32.6 ± 6.9 kg; height 138.8 ± 8.9 cm; BMI 16.8 ± 2.2 kg/m^2^ and %BF (by iDXA) 22.9 ± 8.2%. %BF was strongly correlated with weight (Pearson coefficient, *r* = 0.60) and BMI (*r* = 0.69), and also positively correlated with height (*r* = 0.27; all *p* < 0.001). Onset of puberty (Tanner stage 2) was reported by 34 (13%) children, who were older (mean ± SD: 10.0 ± 0.5 years), heavier (weight: 38.1 ± 8.1 kg; BMI: 18.1 ± 2.7 kg/m^2^) and taller (height: 144.3 ± 6.3 cm) than other children.

### Infancy weight gain associated with later adiposity

In regression models (adjusted for sex and age at follow-up visit), birth weight z-score and changes in weight z-score between 0–3 months and 3–24 months showed independent positive relationships with childhood %BF (Table 2). Effect sizes and standard errors were largest for change in weight z-score over 3–24 months, but partial R-squared values indicated a larger contribution of birth weight z-score and similar contributions of changes in weight z-scores at 0–3 and 3–24 months on later %BF. Together, these variables explained 18.7% of the variation in childhood %BF. The addition of gestational age, parity, or exclusive breastfeeding status at 3 months did not improve prediction accuracy.

**Table 2.** Infant body weight and other predictors of childhood percent body fat (%).

Predictor	Beta	SE	*p* Value	Partial R-squared	Model R-squared	Model AIC
**Baseline model**					18.7%	1746.2
Birth weight z-score	2.51	0.63	<0.001	6.2%		
Change in infancy*:						
weight 0–3 mo	1.51	0.59	0.010	2.7%		
weight 3–24 mo	1.67	0.63	0.007	2.9%		
**Final model**					20.7%	1659.8
Birth weight z-score	2.74	0.64	<0.001	7.2%		
Change in infancy*:						
weight 0–3 mo	1.38	0.69	0.048	1.7%		
weight 3–24 mo	1.71	0.64	0.008	3.0%		
length 0–3 mo	−0.57	0.49	0.244	0.6%		
skinfold thickness 0-3 mo	1.03	0.59	0.086	1.3%		

*Parameterised as a + 1-unit change in z-score during the displayed age period.

Likelihood ratio test comparing baseline versus final models: *p* < 0.0001.

Similarly, birth weight z-score, and changes in weight z-score between 0–3 and 3–24 months showed independent positive relationships with childhood BMI z-score. Partial R-square values indicated a larger contribution of birth weight z-score and similar contributions of changes in weight z-scores at 0–3 and 3–24 months on later BMI (Table 3). Together, these variables explained 13.9% of the variation in childhood BMI z-score.

**Table 3.** Infant body weight and other predictors of childhood BMI (kg/m^2^).

Predictor	Beta	SE	*p* Value	Partial R-squared	Model R-squared	Model AIC
**Baseline model**					13.9%	688.9
Birth weight z-score	0.47	0.08	<0.001	13.1%		
Change in infancy*:						
weight 0-3 mo	0.29	0.07	<0.001	6.1%		
weight 3-24 mo	0.25	0.08	0.001	4.0%		
**Final model**						
Birth weight z-score	0.49	0.08	<0.001	13.7%	14.7%	659.2
Change in infancy*:						
weight 0-3 mo	0.28	0.09	0.002	4.2%		
weight 3-24 mo	0.26	0.09	0.001	4.3%		
length 0-3 mo	−0.07	0.06	0.260	0.6%		
skinfold thickness 0-3 mo	0.10	0.07	0.179	0.8%		

*Parameterised as a + 1-unit change in z-score during the displayed age period.

Likelihood ratio test comparing baseline versus final models: *p* < 0.0001.

### Additional contributions of infancy length and skinfolds

When added to the above regression models for childhood %BF (comprising age at follow-up visit, sex, birth weight z-score, and changes in weight z-score between 0–3 months and 3–24 months), early postnatal changes between 0–3 months in infant length and skinfolds improved overall model prediction, from 18.7% to 20.7% of the variance in childhood %BF (likelihood ratio chi-squared = 90.4, *p* < 0.0001) (Table 2). However, the independent contributions of 0-3 month changes in length z-score (negative trend; partial R-square 0.6%; *p* = 0.2) and skinfolds z-score (positive trend; 1.3%; *p* = 0.09) were small. The addition of birth length, birth skinfold thickness, change in length z-score 3–24 months or change in skinfold thickness z-score 3–24 months did not further improve overall model prediction.

Similarly, when added to the regression models for childhood BMI z-score, early postnatal changes between 0–3 months in infant length and skinfolds improved overall model prediction, from 13.9% to 14.7% of the variance in childhood BMI z-score (likelihood ratio chi-squared = 33.7; *p* < 0.0001) (Table 3). However, again, the independent contributions of 0–3 month changes in length z-score (negative trend; partial R-square 0.6%; *p* = 0.2) and skinfolds z-score (positive trend; 0.8%; *p* = 0.2) were small. The addition of birth length, birth skinfold thickness, change in length z-score 3–24 months or change in skinfold thickness z-score 3–24 months did not further improve overall model prediction.

### Sensitivity analyses

Similar findings were seen when infant weight gain was parameterised as a dichotomous variable “rapid weight gain between 0–24 months” (change in weight z-score ≥ +0.67), although overall model prediction of childhood %BF was, as expected, lower compared to models of continuous change in infancy weight (Supplemental Table 1). Furthermore, findings were similar when pubertal status (yes/no) was added to regression models (Supplemental Table 2).

## Discussion

In this large longitudinal study, with detailed measures of infancy growth parameters from birth to 24 months and childhood adiposity on average 7.5 years beyond infancy, we found that infancy gains in length and skinfolds contributed significantly, but only modestly, to the prediction of childhood adiposity, as estimated by DXA %BF. Similar findings were seen when childhood obesity risk was estimated using BMI, also when infancy weight gain was dichotomised as rapid or non-rapid, and when puberty status was included.

### Strengths and limitations

Strengths of this study are its prospective design, incorporating detailed research clinic measurements of infancy growth parameters beyond only body weight and performed by a consistent team of paediatric nurses with objective validation of the reliability of measures of skinfold thickness at 4 body sites. The primary outcome was assessed by whole body DXA scans, with measures harmonised across two different machines by use of cross-validation data from a large sample of almost 100 children who underwent imaging by both techniques.

We acknowledge a number of limitations. Children attended the childhood clinic visit at a range of ages. This reflected the wide range of birth years of the original cohort and the relatively shorter range of time during which childhood clinic visits were conducted. However, the large majority of children attended between 8–10 years old, and effects of age on the primary outcome were adjusted for. Other more accurate measures of infant adiposity exist, beyond skinfold thicknesses. However, most other such techniques are not easily applicable across ages from birth to 2 years (e.g. PEA POD air displacement plethysmography has a maximum weight of 10 Kg), may require inappropriate restraint or sedation for this age group (DXA), or are not designed for frequently repeated measurements in a large study sample (deuterium labelled water). Furthermore, our recent (unpublished) data show good agreement between infant fat mass predicted by a combination of skinfolds, weight and length, compared to PEA POD measurements. Pubertal status was self-reported, which could introduce some misclassification. However, we consider puberty to be a mediator rather than a confounder (Ong et al. 2009). We used all the available infancy growth data to estimate individual-level growth parameters, but uncertainties in stage one estimation were not subsequently considered when relating these infancy growth parameters to childhood outcomes, and we acknowledge that this approach underestimated the confidence intervals in the final models.

Importantly, we acknowledge that %BF may not be the optimal outcome measure to assess the obesity-related effects of rapid infant growth. Ideally, such studies would benefit from a more robust measure of the burden of obesity-related metabolic disease, such as Type 2 diabetes or carotid intimal thickness during adult life. However, to our knowledge, there are no such studies with long-term follow-up which collected sufficiently detailed measures of infancy growth. Some historical cohorts have reported on very long-term associations between infancy weight and length gain and adult Type 2 diabetes (Eriksson et al. 2003); an important caveat in such long-running studies is to ensure that those populations were not exposed to infancy and early childhood undernutrition or other adverse conditions leading to childhood stunting, which also appears to be separately associated with higher risks for cardiovascular disease and early mortality (Ong et al. 2013), as opposed to the current high nutrition environment which has been shown to extend to the general population of UK infants (Scientific Advisory Committee on Nutrition (SACN) 2018).

Finally, the values for predictive ability in our models were generally low. Infancy prediction of childhood obesity is not in current practice, and therefore, it was not our intention to develop and evaluate a clinical prediction model. We note that such an approach would require an agreed definition of the binary outcome and the models would likely be substantially improved by information on parental BMI (Druet et al. 2012), demographic data and possibly also on infant eating behaviours (Llewellyn and Wardle 2015). However, the potential clinical utility of such approaches is yet unclear (von Kries et al. 2014; Wright et al. 2018).

### Comments on possible biological mechanisms

The Karlberg model of human statural growth (i.e. in length or height) describes 3 phases of childhood growth – infancy, childhood and pubertal – which are distinguished by distinct growth velocities and underpinned by different underlying biological mechanisms (Karlberg 1989). Childhood statural growth is dependent on growth hormone acting to stimulate circulating and tissue production of insulin-like growth factor-1 (IGF-1), and the pubertal growth acceleration is explained by the additional effects of sex steroids, in particular oestradiol in both sexes. By contrast, infancy growth is less reliant on growth hormone, but rather IGF-1 levels more reflect level of nutrition acting via insulin secretion (Ong et al. 2007). This mechanistic understanding provides an explanation as to why overnutrition may have unique effects during infancy on promoting statural growth and the substantial accumulation of fat-free mass, alongside positive effects on fat mass that are typical of all other age periods.

Recent population genetics studies support the concept that infancy overnutrition has uniquely global effects across both fat and fat-free tissues. The genetic susceptibility to higher adult BMI has been extensively studied in large international collaborative efforts, which have led to the discovery of hundreds of individual genomic loci that are each robustly associated with adult BMI (Locke et al. 2015). The genetic susceptibility captured by the additive combination of these variants has been shown to be relevant to obesity risk at all ages, and confers higher childhood and adult adiposity, and higher risks of the cardiometabolic disease consequences of obesity. These genes predominantly appear to act through central mechanisms of appetite regulation and eating behaviours (Llewellyn and Wardle 2015; Locke et al. 2015; de Lauzon-Guillain et al. 2017), with effects being apparent in infants as well as in adults (Llewellyn et al. 2012; Llewellyn and Wardle 2015; de Lauzon-Guillain et al. 2019). Consistent with the idea that the genetic susceptibility to obesity acts by promoting appetite and food intake throughout life, additive genetic risk scores comprising these variants have been shown to confer a faster trajectory of weight gain from birth through childhood, with possibly even larger effect sizes during infancy than in other periods of life (Elks et al. 2012, 2010). In support of the current findings and in keeping with the Karlberg model of growth, during infancy, higher BMI genetic risk scores confer faster symmetrical gains in infant weight and length, as well as similar size gains in infant fat and fat-free mass – whereas, effects on indices of weight-for-height (e.g. BMI) and on adiposity relative to fat-free mass (e.g. %BF) start to appear only on transition to the childhood growth phase (Elks et al. 2014). Hence, the Karlberg model (Karlberg 1989) provides biological plausibility for the current findings, that infancy length and adiposity contribute only modestly when added to infancy weight to predict later predisposition to obesity.

We note, as a caveat, that our findings do not exclude the possibility that infancy length and adiposity (and also infant body fat distribution) might contribute more to the prediction of later metabolic parameters (e.g. glucose tolerance, insulin resistance) and related disease endpoints (Type 2 diabetes, cardiovascular disease) than to later body composition. For example, we have previously described phenotypic and genetic links between childhood height and insulin secretion (Ong et al. 2004; Jensen et al. 2015), likely mediated by insulin-like growth factor-1. Furthermore, recent population genetic studies demonstrate the causal relationship of central versus peripheral fat patterning on such metabolic and disease traits (Lotta et al. 2017), and such fat patterning is already apparent in infancy (Breij et al. 2017).

## Conclusion

Infancy length and skinfolds contributed significantly, but only modestly, to the prediction of childhood adiposity, as estimated by DXA %BF. Hence, in infants who display rapid weight gain, similar gains in length and body composition do not appear to substantially modify their subsequent higher risk of later obesity. These findings are consistent with the widely held biological concept of an underlying nutritional regulation of infancy growth and body composition. The possible clinical utility of infancy prediction of childhood adiposity requires a much broader consideration, including wider infancy and parental data, and appropriate outcome measures and their thresholds.

## Supplementary Material

SUPPLEMENTAL

## Data Availability

The data that support the findings of this study are not available for replication outside members of the research group due to conditions of participant consent. Anonymized data are available to other investigators through collaborative agreements, and we welcome proposals to collaborate on related projects using this dataset.
