# Supplementary material for: Which infancy growth parameters are associated with later adiposity? The Cambridge Baby Growth Study
Source: Ann Hum Biol. 2020 May 20;47(2):142–9. doi: 10.1080/03014460.2020.1745887 (PMC7261401; doi:10.1080/03014460.2020.1745887)
Supplement: SUPPLEMENTAL [file IAHB_A_1745887_SM1131.pdf]

Supplementary Figure 1. Flow diagram describing identification of the included study sample

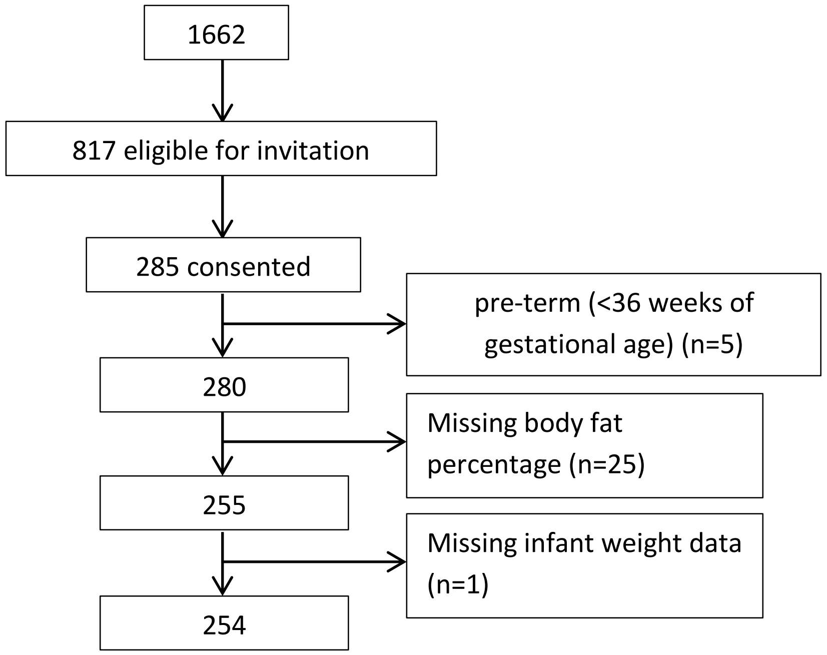

Supplementary Figure 2. Smoothed plots of average predicted infancy growth

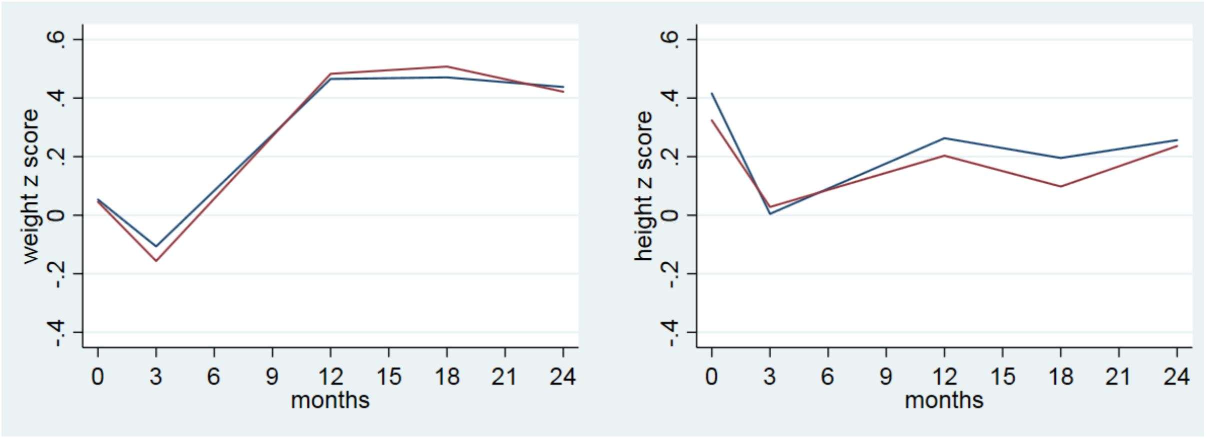

Supplementary Table 1: Birth weight, rapid infancy weight gain and other predictors of childhood percent body fat (%)

Predictor	Beta	SE	*P value*	Partial R-squared	Model R-squared	Model AIC
**Basic model**					17.5%	1754.5
Birth weight z-score	1.98	0.54	<0.001	5.1%		
Rapid weight gain 0-24 mo	2.49	1.05	0.019	2.2%		
**Final model**					19.5%	1661.4
Birth weight z-score	2.28	0.58	<0.001	6.3%		
Rapid weight gain 0-24 mo	2.35	1.12	0.037	1.9%		
Change in infancy*:						
length 0-3 mo	-0.45	0.47	0.336	0.4%		
skinfold thickness 0-3 mo	1.13	0.57	0.048	1.7%		

*Parameterised as a +1-unit change in z-score during the displayed age period
Likelihood ratio test comparing baseline versus final models: *P*<0.0001

Supplementary Table 2: Final models for prediction of childhood percent body fat or BMI* with adjustment for pubertal status

Predictor	Beta	SE	*P value*	Partial R-squared	Model R-squared	Model AIC
**Childhood percent body fat (%)**					18.8%	1463.5
Birth weight z-score	2.63	0.67	*<0.001*	7.1%		
Change in infancy*:						
weight 0-3 mo	1.40	0.71	*0.048*	1.9%		
weight 3-24 mo	1.27	0.67	*0.059*	1.7%		
length 0-3 mo	-0.64	0.51	*0.213*	0.8%		
skinfold thickness 0-3 mo	0.83	0.62	*0.181*	0.9%		
**Childhood BMI (kg/m^2)**					15.4%	586.3
Birth weight z-score	0.44	0.09	*<0.001*	11.4%		
Change in infancy*:						
weight 0-3 mo	0.31	0.09	*0.001*	5.3%		
weight 3-24 mo	0.21	0.09	*0.018*	2.7%		
length 0-3 mo	-0.09	0.07	*0.195*	0.8%		
skinfold thickness 0-3 mo	0.05	0.08	*0.568*	0.2%		
**Childhood percent body fat (%)**					18.0%	1463.6
Birth weight z-score	2.21	0.61	*<0.001*	6.1%		
Rapid weight gain 0-24 mo	2.06	1.15	*0.076*	1.5%		
Change in infancy*:						
length 0-3 mo	-0.44	0.49	*0.370*	0.4%		
skinfold thickness 0-3 mo	1.02	0.58	*0.081*	1.5%		

**Childhood lean mass index (kg/m²)**					12.0%	-3256.2
Birth weight z-score	2.3E-5	1.0E-5	*0.026*	2.4%		
Change in infancy*:						
weight 0-3 mo	2.3E-5	1.1E-5	*0.043*	2.0%		
weight 3-24 mo	1.1E-5	1.0E-5	*0.276*	0.6%		
length 0-3 mo	-1.5E-6	8.0E-6	*0.850*	0.02%		
skinfold thickness 0-3 mo	-6.7E-6	9.7E-6	*0.491*	0.2%		

*Parameterised as a +1-unit change in z-score during the displayed age period
